# Evaluation of the Effects of Caffeic Acid Phenethyl Ester on Prostaglandin E_2_ and Two Key Cytokines Involved in Bleomycin-induced Pulmonary Fibrosis

**Published:** 2013-07

**Authors:** Amir Larki-Harchegani, Ali Asghar Hemmati, Ardeshir Arzi, Mehri Ghafurian-Boroojerdnia, Somayeh Shabib, Mohammad Reza Zadkarami, Saleh Esmaeilzadeh

**Affiliations:** 1 Department of Pharmacology and Toxicology, School of Pharmacy and Physiology Research Center, Jundishapur University of Medical Sciences, Ahvaz, Iran; 2 Department of Immunology, School of Medicine, Jundishapur University of Medical Sciences, Ahvaz, Iran; 3 Department of Statistics, School of Mathematics and Computer Sciences, Shahid Chamran University, Ahvaz, Iran; 4 Department of Pathobiology, School of Veterinary Medicine, Shahid Chamran University, Ahvaz, Iran

**Keywords:** Bleomycin, CAPE, Cytokine, Pulmonary Fibrosis, PGE2, TGF-β1, TNF-α

## Abstract

***Objective(s):*** Pulmonary fibrosis (PF) is the most common outcome of a collection of diverse lung disorders known as interstitial lung diseases. It is proposed that alterations in the levels of fibrogenic mediators and the profibrotic/antifibrotic imbalance play a substantial role in the progression of PF in animal models and possibly in humans. Caffeic acid phenethyl ester (CAPE), an active component of propolis, has numerous biological effects. In the present study, the main objective was to investigate the effects of CAPE on some key mediators including TGF-β_1_, TNF-α and prostaglandin E_2 _(PGE_2_) involved in profibrotic/antifibrotic balance and pathogenesis of idiopathic pulmonary fibrosis (IPF).

***Materials and Methods: ***In this study, forty male Sprague–Dawley rats were divided into 5 groups (n=8). (1) “Bleomycin (BLM)-treated (Model) group”: BLM (5 mg/kg, single intratracheal dose), (2) “Saline-treated group”: the rats were given only saline, (3) “Treatment-1 group”: BLM + CAPE (5 μmol/kg/day, 28 days, IP), (4) “Treatment-2 group”: BLM + CAPE (10 μmol/kg/day, 28 days, IP) and (5) “Vehicle + CAPE group”: CAPE (10 μmol/kg/day, 28 days, IP).

***Results:*** BLM could significantly increase the levels of TNF-α and TGF-β_1_ and decrease the PGE_2_ concentration compared to the saline control group. CAPE could considerably improve these values almost close to normal levels.

***Conclusion:*** Briefly, CAPE can be suggested as a novel, attractive and effective agent for prevention and treatment of pulmonary fibrosis.

## Introduction

Pulmonary fibrosis (PF) is a common final consequence of diverse lung injuries with different etiology and pathologic features which is usually resulted from dust inhalation, radiation, drugs use, chemicals with pulmonary toxic properties, and also some systemic and pulmonary diseases (1). Idiopathic pulmonary fibrosis (IPF) also known as cryptogenic fibrosing alveolitis (CFA) is limited to the lungs and is typically associated with a histopathological pattern of usual interstitial pneumonia (UIP) (-). In IPF, fibrosis is typically caused by an early severe alveolitis in which a set of complex interactions occurs between various mediators and cell types including epithelial, endothelial, inflammatory and fibroblastic cells. Eventually, it is the severity of alveolitis which defines the speed and severity of disease progression. 

Epithelial and endothelial cells injury seems to be the initial event in IPF. This argument is confirmed by hyperplasia of type II cells and proteinaceous exudate found in the alveolar air spaces (5). Epithelial cells produce endothelin-1, profibrotic and proinflammatory cytokines and tissue factors that lead to deposition of fibrin in the alveolar spaces (6, 7). Endothelial damage also plays a critical role in the pathogenesis of IPF. Endothelial damage promotes collagen deposition in the interstitium by activating the attractant effect of thrombin on fibroblasts (3). A growing body of scientific evidence indicates that polypeptide mediators known as cytokines are one of the most important factors involved in collagen deposition during pulmonary fibrosis. A large number of cytokines are involved in the thogenesis of IPF including transforming growth factor-β (TGF-β)  (8), tumour necrosis factor-α (TNF-α) (9), insulin-like growth factor-1 (IGF-1) (10), endothelin-1 (ET-1) (11), interleukin (IL)-1, IL-7 (12), IL-8 (13), IL-10  (8) and IL-12 (14). Prostaglandins (PGs) are members of the eicosanoid family of signaling molecules involved in a number of physiological processes in many tissues. PGE_2 _is the most ubiquitously produced isoform of prostaglandin subset and a potent inhibitor of fibroblast proliferation, collagen synthesis, and fibroblast to myofibroblast differentiation (15) . In the presence of increased TGF-β_1_ levels in the lung, PGE_2_ deficiency could thus lead to increased fibroblast proliferation and thereby enhancing the fibrotic response. The widespread use of BLM in animal models of pulmonary fibrosis is based on the fact that fibrosis is one of the major adverse effects of BLM in human cancer chemotherapy. Therefore, BLM has become one of the most commonly used agents in the study of the pathogenesis of human idiopathic pulmonary fibrosis because it can reproducibly cause a similar condition in experimental animals. 

CAPE is the most important biologically active ingredient of bee propolis extract. This polyphenolic lipophilic flavonoid natural product has numerous biological activities such as antioxidant (16) anti-cancer, anti-viral (17), antitumor (18, 19), anti-inflammatory (20) and immunomodulatory properties (21). Considering the beneficial effects of CAPE observed in previous studies, the present study was designed to investigate the effects of CAPE on rat pulmonary fibrosis model focusing particularly on a number of factors involved in the profibrotic/antifibrotic balance.

## Material and Methods

***Animals***

A total of 40 pathogen-free male Sprague–Dawley (SD) rats weighing 180–220g were obtained from the Laboratory Animals Care, Breeding and Research Center, Jundishapur University of Medical Sciences, Ahvaz, Iran. The animals were randomly divided into five groups and kept in polypropylene cages (dimensions: 55 cm 35 cm 22 cm) on hardwood shavings with a maximum of six rats in each cage. They were housed in a temperature-controlled room (23±2°C) with 12 hr light/dark cycles and had free access to standard rat chow and water. The rats were allowed to acclimate in animal house facilities of pharmacology laboratory for at least 1 week before any treatment. They were weighed on day 0, 7, 14, 21 and at the time of sacrifice. All animal experiments were performed in accordance with the principles of the National Institutes of Health for experimental care and use of animals.

***Chemical agents***

Bleomycin sulfate (BLEO-S^®^) was obtained from NIPPON KAYAKU CO. (Tokyo, Japan). Caffeic acid phenethyl ester was purchased from Sigma, St. Louis, MO, USA. The rat TNF-α and TGF-β_1_ ELISA kits were obtained from eBioscience company (San Diego, CA, USA). Rat Prostaglandin E_2_ (PGE_2_) ELISA kit was obtained from CUSABIO BIOTECH Co. (Wuhan University Science Park, Wuhan, Hubei Province 430223, P.R.China). All the other chemicals used in this study were of the highest grade commercially available.

***Drug treatment groups***

The animals were randomly assigned to one of the five experimental groups (n=8 for each group) as follows: 

(1) “Positive control (Model) group”: animals were treated with a single intratracheal (IT) dose of BLM solution (5 mg/ml/kg of body weight), BLM solution was freshly prepared in saline and the concentration was adjusted so that each animal received 0.1 ml/100 g body weight (22).

(2) “Negative control group”: rats were given only a single IT dose of saline (maximum permissible volume = 1 ml/kg) (23).

(3, 4) “Treatment groups 1 and 2”: the rats in these groups received CAPE at the dose of 5 and 10 μmol/kg/day, respectively, twice a day for 28 days, starting 7 days before induction of lung fibrosis by a single IT dose of BLM (5 mg/kg/ml) and continued for 21 days until the end of the experiment (23).

(5) The effects of CAPE 10 μmol/kg plus vehicle (without BLM) was considered as “sham group” to ensure that higher dose of CAPE (10 μmol/kg) tested in this study is safe and has no toxic effect on the lung tissue of the animals. On the 7th day, BLM or saline was administered intratracheally. The CAPE doses were given intraperitoneally (IP) twice daily starting from the day 0 and continued for 4 weeks. Saline was used as the vehicle in preparation of all solutions.

***Induction of pulmonary fibrosis by bleomycin***

 Based on the method explained by Schraufnagel *et- al* (1986), rats were anaesthetized with ether, then placed on a slanted board and hanged from their upper incisors. Keeping the nose of animal closed and its tongue out, BLM solution (5 mg/kg) was delivered via the mouth into the trachea by a modified needle at the maximum volume of 1 ml/kg body weight. Rats in the saline-treated group received intratracheal instillation of the same volume of saline (1 ml/kg body weight). After recovery from anesthesia, rats were returned to their cages (22).

***Isolation of lung tissue samples and serum sampling***

At the end of the treatment course, all rats were weighed, blood sampling was performed under light ether anaesthesia by cardiac puncture (24), and the serum was extracted immediately. To avoid repeated freeze-thaw cycles, the serum sample of each animal was divided into several aliquots (about 0.5 ml each). The serum samples were stored at -70°C for the next steps of the study. As soon as blood sampling was done, the animals were killed with a lethal dose (120 mg/kg, IP) of sodium pentobarbitone (Sagatal®). After mid-line sternotomy, whole lung (including both lobes) was dissected out, separated from other tissues, washed free of blood with ice-cold saline, and placed in a sterile plastic petri dish. Then, the right lobe of the animal lungs were preserved in buffered formaldehyde solution (10% w/v) for histopathological assessment. To prepare lung tissue homogenate samples, the left lobe of animal lungs were excised, rinsed with ice-cold saline solution, and quickly frozen in liquid nitrogen before being stored at -70 °C. Later, the frozen left lungs were thawed, and approximately 500 mg of each tissue sample was quickly homogenized in 5 ml of PBS (1X) to yield a 10% *w/v* tissue homogenate and then stored at -20°C, overnight. Two freeze-thaw cycles were performed to break the cell membranes. Then, the homogenates were centrifuged for 5 minutes at 5000g. The supernatant was immediately removed, aliquoted, and stored at -70° C for biochemical assay.

***Biochemical and immunological assays***

In different study groups, the contents of PGE_2_ (an eicosanoid with antifibrotic effects) in lung tissue homogenate and the concentrations of TNF-α and TGF-β_1 _(as proinflammatory and profibrotic cytokines) in serum samples were analyzed by ELISA using commercially available kits according to the manufacturer’s instructions. The samples were finally read by ELISA reader.

***Collagen assay***

Collagen content of lung tissue was measured as an index of the extent of lung tissue fibrosis. In this study, we used a simple quantitative micro-assay tool for determining the amounts of collagen and non-collagenous proteins in tissue sections by differential staining with two dyes; Sirius Red and Fast Green. The tissue samples were cut with a razor blade and immediately fixed in 10% formalin, then, they were embedded in paraffin and sections of approximately 15 µm thicknesses were obtained. Given the patchy distribution of bleomycin-induced fibrosis throughout the lung, it was necessary to generate a measure of total lung collagen. This can be achieved by measuring collagen from the whole lung. For this reason, multiple sections (about 30 sections) were sampled from different levels in the lung and the mean of collagen content in these sections was calculated to generate the biochemical and histological data. Approximate amount of collagen in lung tissue of each animal (X) can be achieved using the following equation:

X=(animal lung weight)×(mean of collagen content in 30 sections)(mean weight of 30 sections)

The evaluation of collagen content was based on the method published by Lopez de Leon and Rojkind (25). 

***Histological evaluation and scoring***

The lung tissue sections were stained either with hematoxylin and eosin (H&E) to visualize tissue architecture or with Masson’s trichrome stain to detect collagen deposits. Histopathological evaluation is a current gold standard for diagnosis and staging of IPF. Therefore, a reliable scoring system for the assessment of lung fibrosis is necessary. Ashcroft *et al*. approached this problem by assigning a numerical scale, from 0 to 8, of the amount of fibrotic tissue in histological samples. This scale was replaced by Ralf-Harto Hübner *et al* (2008) scale (the Ashcroft modified scale) due to a considerable degree of variability between the results obtained from different research laboratories which made the comparisons difficult (26). The structural alterations and the degree of fibrosis in lung specimens during the study were assessed and rated by two independent certified pathologist blinded to the study groups using Ashcroft modified scoring system (26). All slides were scored from 0 to 8 according to the degree of cellular proliferation, alveolar wall thickening, inflammatory lesions and collagen deposition or fibrosis.

***Statistical analysis***

Data were analyzed using a one-way analysis of variance (ANOVA) followed by a post hoc test (Tukey’s test). Fibrosis scores of lung tissue were evaluated using Mann–Whitney test. Statistical analysis was performed using SPSS software, and the probability values of 0.05 or less were considered statistically significant. The results were expressed as means ± SEM (Standard Error of Mean) for eight rats per each experimental group.

## Results

**Figure 1:**
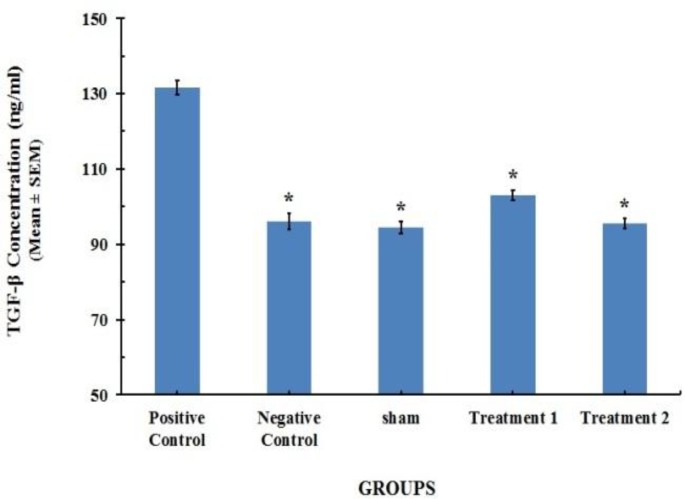
TGF-β_1_ content in serum specimens of the rats (n=8). Each value represents Mean±SEM. Significant difference versus positive control group has been shown by * (*P*<0.001).

**Figure 2:**
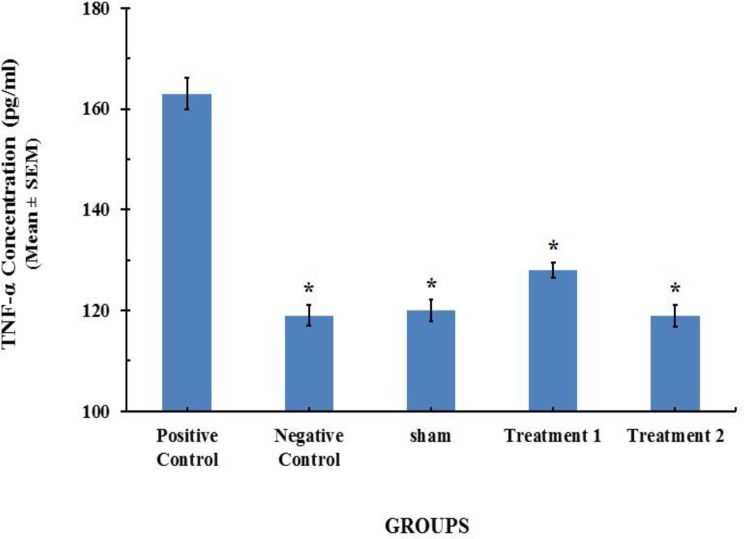
TNF-α content in serum of the rats (n=8). Each value represents Mean±SEM. Significant difference versus positive control group has been shown by * (*P*<0.001).

***TGF-β***_1_ ***and TNF-α serum concentration profile ***Twenty-one days after BLM instillation in the model group, the level of TGF-β_1_ and TNF-α in serum significantly increased on day 21, compared with [96.01 ± 0.74 (ng/ml)] and [163±1.13 (pg/ml)] (*P*< 0.001) for saline control group, respectively. CAPE at the dose of 5 (µM/kg/day) could reduce the serum levels of TGF-β_1_ and TNF-α to [103.02 ± 0.48 (ng/ml)] and [128±0.53 (pg/ml)], respectively. However CAPE at the dose of 10 (µM/kg/day) could decrease concentrations of TGF-β_1_ and TNF-α more than lower dose of 5 (µM/kg/day). The results are shown in Figure 1 and 2.

**Figure 3:**
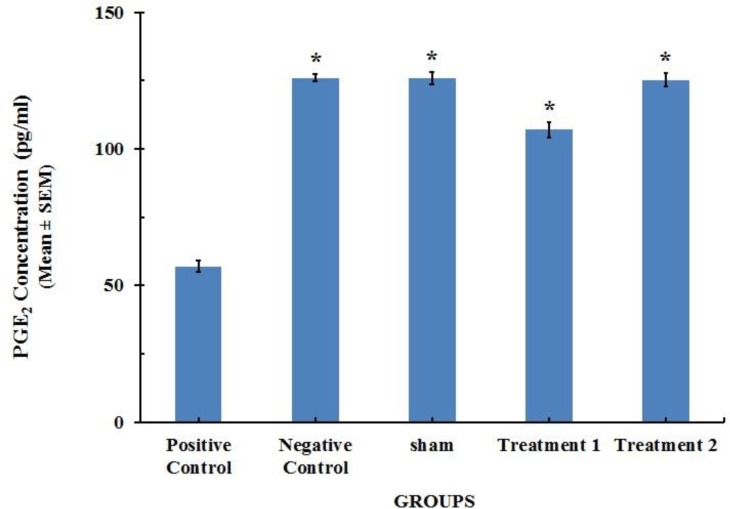
PGE_2_ content in lung tissue homogenate specimens of the rats (n=8). Each value represents Mean±SEM. Significant difference versus positive control group has been shown by * (*P*<0.001

**Figure 4:**
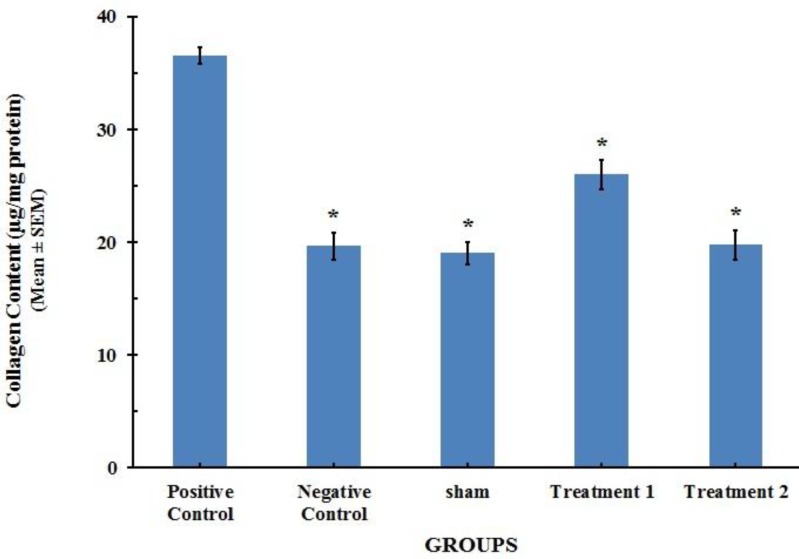
Collagen content in lung tissue of the rats (n=8). Each value represents Mean±SEM. Significant difference versus positive control group has been shown by * (*P*<0.001).

***PGE***_2_ ***lung tissue concentration profile***

The concentration of prostaglandin E_2_ in lung tissue was significantly decreased by BLM administration on 28^th^ day of the study. This effect was abrogated by CAPE treatment which increased the level of PGE_2_. However, the effect of CAPE at the dose of 10 (µM/kg) on the levels of PGE_2 _was more pronounced compared with a lower dose of CAPE (5 µM/kg). There were no significant differences in the PGE_2_ concentrations between negative control group and Sham group showing that the systemic administration of CAPE was safe (Figure 3).

***Collagen assay***

The collagen content in the lung tissue of positive control group was markedly increased in comparison with the value of the negative control group (*P*<0.001), suggesting enhanced tissue fibrotic activity as compared with the saline control group. This increase in the lung collagen content induced by BLM was attenuated with CAPE treatment to the normal levels that were close to control values (Figure 4).

**Histopathological results**

**Figure 5:**
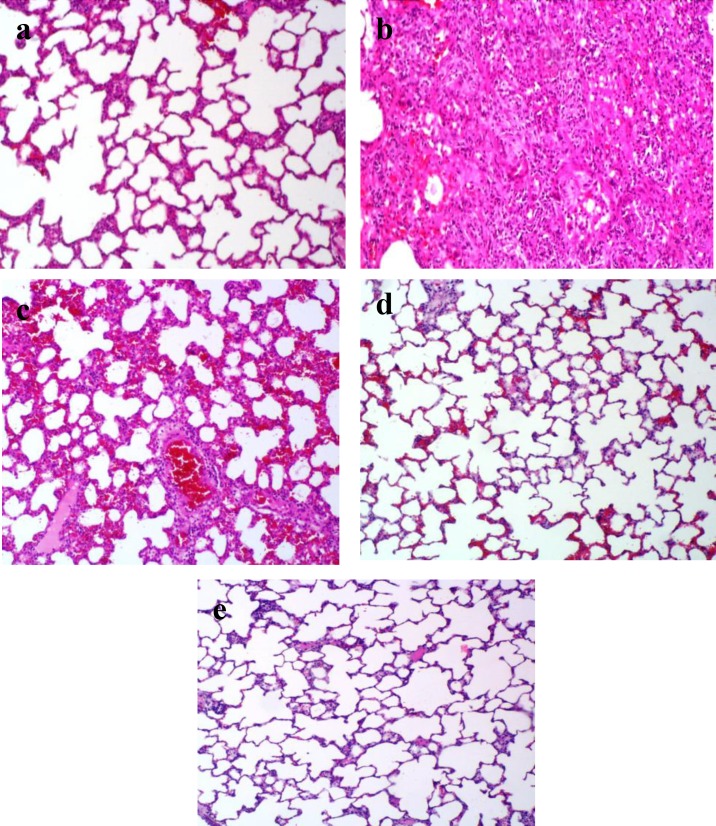
Photomicrographs of lung tissue section of the rats in the study groups. Original magnification of all images is ×10. , (a) Negative control group, (b) positive control group, (c) BLM + CAPE (5 µmol/kg) group, d) BLM + CAPE (10 µmol/kg) group, (e) Sham group

At the end of the study course, a morphological evaluation of the lung tissue sections was performed by light microscopy for the 5 different study groups. The histological analysis showed that the lungs of rats in negative control group had normal alveolar spaces, normal alveolar septa, normal lung structure, and no lesion was evident (Figure 5.a). Three weeks after intratracheal administration of BLM, in positive control group, there were increased infiltration of inflammatory cells, collapse of alveolar spaces and diffused damage to lung architecture, which clearly indicated typical pulmonary fibrosis (Figure 5.b). In CAPE-treated groups, lungs were effectively protected against tissue damage caused by BLM toxicity. In these groups, fewer inflammatory cells and decreased alveolar thickening was evident. However, in different treatment groups, the severity of changes varied from slight to moderate (Figure 5.c & 5.d).

***Ranking the severity of lung injury***

**Figure 6:**
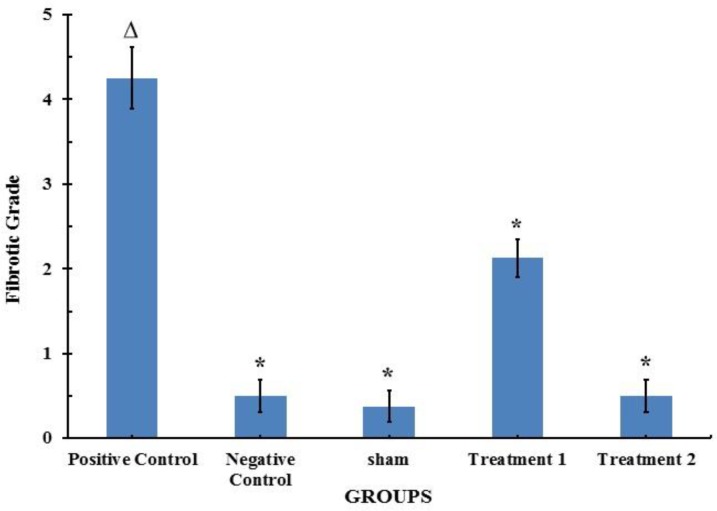
Effect of CAPE on BLM-induced histological changes in the lung tissue of the rats (n=8). Each value represents Mean±SEM. Significant difference versus positive and negative control groups is shown by * and ∆, respectively (*P*< 0.001

The fibrosis score was significantly higher in the lungs of BLM-treated rats, compared with the negative control group (*P*<0.001). Consistent with the results of collagen assay, CAPE could significantly reduce the BLM-induced promotion in severity (score) of fibrosis (Figure 6).

## Discussion

Pulmonary fibrosis (PF) is a progressive lung disorder characterized by accumulation of extracellular matrix (ECM) proteins (27). Unfortunately, despite its high impact on human health, no effective treatment has been yet developed. In this disorder, the inflammatory cells including alveolar macrophages act through releasing mediators such as eicosanoid metabolites, destructive proteolytic enzymes and inflammatory growth and differentiation factors. It is likely that dysregulations in the balance of some growth factors play major roles in determining the differences between normal and pathologic tissue repair. Among these, TGF-β is one of the key cytokines involved in the pathogenesis of pulmonary fibrosis (28). Several publications investigating tissue fibrosis have focused on the most prominent isoform, TGF-β_1_, demonstrating an array of profibrotic functions. It is well established that TGF-β_1_ promotes differentiation of fibroblasts into activated myofibroblasts, enhances collagen synthesis, and reduces collagen degradation by down-regulation of proteases and up-regulation of protease inhibitors (29). Early studies have also revealed that expression of TGF-β_1_ is up-regulated in BLM-induced animals (30, 31). In our study, the pathological changes in the lung tissue caused by BLM were consistent with the findings of other studies (26, 32). This early result can be an explanation for the down-regulation of TGF-β_1_ and the subsequent reduction of collagen accumulation in the lung tissue of CAPE treated groups. Our results revealed that CAPE evidently attenuated subsequent collagen deposition. 

TNF-α is produced by many different cell types including stimulated monocytes, fibroblasts and endothelial cells which are the main source of TNF-α, *in vivo*. TNF-α can stimulate fibroblast replication and collagen synthesis *in vitro*, and pulmonary TNF-α gene expression rises after administration of BLM in mice (33) . In a study conducted by Chen *et al*, reduced fibrosis in animals given anti-mouse TNF-α (ATCC HB-10649) Monoclonal Antibody (mAb) correlated with reduced expression of active TGF-β and MCP-1 (Monocyte Chemotactic Protein-1) and decreased infiltration of myofibroblasts. Anti-TNF-α also markedly suppressed BLM-induced lung fibrosis and decreased the expression of TGF-β and MCP-1 in the lung. Anti-TNF-α may inhibit fibrosis through down-regulation of profibrotic cytokines such as TGF-β and MCP-1 (34). On the other hand, TGF-β_1_ is a major fibrogenic cytokine involved in the development of pulmonary fibrosis. Thus, it can be said these two relevant cytokines (TGF-β_1_ and TNF-α) contribute to airway fibrosis because of their ability to regulate fibroblast and matrix production (35). In other words, TGF-β_1_ as a link between inflammation and fibrosis is considered to promote lung structure changes (36).

It has been reported that PGE_2_ is a potent inhibitor of fibroblast proliferation, collagen synthesis, and fibroblast to myofibroblast differentiation (15). Therefore, the failure of PGE_2_ synthesis has been shown to be associated with a decreased capacity to up-regulate cyclooxygenase 2 (COX-2) (-).

A growing body of evidence supports the hypothesis that PGE_2_ has a crucial role in the modulation of tissue repair and lung fibrosis (40). PGE_2_ inhibits fibroblast migration and proliferation in response to various mitogens and abrogates TGF-β induced collagen production (41, 42). In addition, fibrotic fibroblasts exhibit a marked reduction in the ability to up-regulate PGE_2_ synthesis in response to TGF-β, with the consequent loss of the antiproliferative response to TGF-β mediated by PGE_2 _(43). Moreover, lung epithelial cells are a major source of PGE_2_, and the capacity of these cells to inhibit fibroblast proliferation is related to their ability to produce PGE_2 _(44).

Nowadays given the identification of various cytokines, growth factors and other mediators involved in fibrosis process, it seems reasonable to develop agents which can potentially modulate the activity and amount of cytokines and mediators, concommitantly. 

CAPE, a potent flavonoid anti-oxidant, has strong anti-viral, anti-tumoral, anti-inflammatory, anti-oxidant, neuroprotective, anti-atherosclerotic and immunomodul-atory properties in diverse systems (45).

Our results showed that in BLM model group, the concentrations of TNF-α and TGF-β_1_ were significantly increased in comparison with the vehicle treated rats. In fact, CAPE particularly at the dose of 10 (µM/kg/day), could decrease concentrations of these key cytokines (TNF-α and TGF-β_1_) close to their normal values.

One of the most relevant findings in our study was the effect of CAPE on PGE_2_ synthesis. CAPE significantly abolished the decrease in PGE_2_ synthesis induced by BLM. Our results confirmed that CAPE increases PGE_2_ and reduces collagen deposition. The PGE_2_ tissue concentrations were determined in lungs of different groups after completion of the treatment course.

Taken together, these observations indicate that CAPE acts on at least two of the most crucial mediators implicated in lung fibrosis, improving the balance between profibrotic (TGF-β_1_) and antifibrotic anti-fibrotic (PGE_2_) mediators.

Histopathological observations in this study confirm the results obtained in other studies (46-48), and in these studies CAPE (10 μmol/kg) could represent a well protection against the pathological alterations caused in models of PF.

Therefore, we deduced that CAPE may be able to improve pulmonary fibrosis not only because of it’s ability to decrease expression and activation of some inflammatory and fibrogenic cytokines, but also it alleviated multi-interactions among them.

NF-κB induces the expression of a wide variety of genes involved in inflammation and fibrosis, including encoding cytokines (such as TNF-α), enzymes (including nitric oxide synthase), adhesion molecules and acute-phase proteins. CAPE is also a potent and specific inhibitor of nuclear transcription factor-κB (NF-κB) activation (46). 

Zhang *et al* investigated the role of TNF-α in pulmonary fibrosis induced by BLM. They suggested a novel mechanism via which TNF-α could mediate pulmonary fibrosis through induction of IL-5-mediated eosinophil recruitment and fibrogenic cytokine production. This cytokine networking which is orchestrated by TNF-α including TGF-β_1_ and other fibrogenic mediators, which in turn, amplifies the inflammatory response and drives the progression to fibrosis and end-stage lung disease (47). In fact, by inhibiting the nuclear transcription factor-κB (NF-κB) activity, CAPE interrupts the synthesis of key mediators such as TNF-α which trigger cascade of mediators contributing to fibrogenic process including TGF-β_1_ and other fibrogenic and inflammatory mediators.

The relevance of COX-2 as a protective mediator of pulmonary fibrosis has been demonstrated *in vivo* and in vitro (43, 48, 49). COX-2 is the major source of the PGE_2_ synthesized by alveolar epithelial cells (50). The failure of PGE_2_ synthesis in fibroblasts and lung tissue from patients with IPF has been shown to be associated with a decreased capacity of COX-2 up-regulatation. (37, 43, 51)

Interestingly, according to the study conducted by Michaluart *et al*. (52), CAPE has inhibitory effects on the activity and expression of cyclooxygenase-2 in human oral epithelial cells and in a rat model of inflammation. Thus, CAPE probably increases the synthesis of PGE_2 _through mechanisms other than upregulation of COX-2. In another study done by Molina (2006) (53), increased PGE_2_ level observed in lung fibrosis following losartan treatment did not correlate with a significant increase in COX-2 mRNA expression. The author concluded this could be a consequence of other enzymes such as PGE_2_ synthase which is implicated in PGE_2_ synthesis. However, in our study the histopathological results appear to be consistent with the Molina's hypothesis. In other words, increased PGE_2_ synthesis was probably due to the increased activity of other enzymes including PGE_2_ synthase.

## Conclusion

Taking together, the results of this study clearly indicate that BLM can cause evident pulmonary fibrosis and significant increase in the fibrotic grade shortly after intratracheal administration to the animals. However, data reported here reveal that CAPE can dose-dependently attenuate bleomycin-induced lung fibrosis and pulmonary damage in rats. This improvement is associated with decreased collagen deposition and pathological grade. The underlying mechanisms of the good protective effect of CAPE may be attributed to modulation of TGF-β_1_, TNF-α and PGE_2_ levels, decrease in collagen content, improvement of pathologic changes, and decrease in the fibrotic grade in BLM-induced pulmonary fibrosis in rat. Therefore, CAPE could be considered as a valuable novel agent for prophylaxis or treatment of pulmonary fibrosis.
